# A Covalently Cross-Linked Hyaluronic Acid/Carboxymethyl Cellulose Composite Hydrogel as a Potential Filler for Soft Tissue Augmentation

**DOI:** 10.3390/gels10010067

**Published:** 2024-01-16

**Authors:** Francesca Della Sala, Mario di Gennaro, Pooyan Makvandi, Assunta Borzacchiello

**Affiliations:** 1 Institute of Polymers, Composites and Biomaterials, National Research Council (IPCB-CNR), Viale J.F. Kennedy 54, 80125 Naples, Italy; francesca.dellasala@cnr.it (F.D.S.); mariodigennaro5@gmail.com (M.d.G.); 2 Centre of Research Impact and Outcome, Chitkara University Institute of Engineering and Technology, Chitkara University, Rajpura 140401, Punjab, India; pooyanmakvandi@gmail.com

**Keywords:** hyaluronic acid, carboxymethyl cellulose, BDDE, hydrogels, fillers

## Abstract

The use of fillers for soft tissue augmentation is an approach to restore the structure in surgically or traumatically created tissue voids. Hyaluronic acid (HA), is one of the main components of the extracellular matrix, and it is widely employed in the design of materials with features similar to human tissues. HA-based fillers already find extensive use in soft tissue applications, but are burdened with inherent drawbacks, such as poor thermal stability. A well-known strategy to improve the HA properties is to reticulate it with 1,4-Butanediol diglycidyl ether (BDDE). The aim of this work was to improve the design of HA hydrogels as fillers, by developing a crosslinking HA method with carboxymethyl cellulose (CMC) by means of BDDE. CMC is a water soluble cellulose ether, whose insertion into the hydrogel can lead to increased thermal stability. HA/CMC hydrogels at different ratios were prepared, and their rheological properties and thermal stability were investigated. The hydrogel with an HA/CMC ratio of 1/1 resulted in the highest values of viscoelastic moduli before and after thermal treatment. The morphology of the hydrogel was examined via SEM. Biocompatibility response, performed with the Alamar blue assay on fibroblast cells, showed a safety percentage of around 90% until 72 h.

## 1. Introduction

Disease, trauma, and aging result in the loss of dermal collagen and fat, leading to deficits in soft tissue [1]. As a consequence, there is a need to develop materials that safely and effectively restore areas of deficiency. Soft tissue fillers have been used for decades for reconstructive and aesthetic procedures [2]. HA is a naturally occurring biopolymer, mainly concentrated in soft connective tissue extracellular matrix (ECM), dermis, vitreous body of the eye, hyaline cartilage, synovial joint fluid, intervertebral disc nucleus pulpous, and umbilical cord. HA consists of repeating disaccharide units composed of N-acetyl-D-glucosamine and D-glucuronic acid linked by a β-1,4 glycosidic, bond, whereas the disaccharides are linked by β-1,3 glycosidic bonds [3]. Its unique characteristics, such as biocompatibility, biodegradability, and mucoadhesiveness, as well as its viscoelastic properties, have led it to be used in a versatile manner in various biomedical applications [4]. HA-based hydrogels hold great promise for soft tissue engineering to replace damaged or lost tissues, since these biomaterials provide an environment close to the native ECM. It has been found that HA-based filler materials are useful in corrective and surgical fields, for example in aesthetic applications (such as facial contouring and in products for soft tissue augmentation), surgery, such as in sutures, drug administration, and moist wound dressing [5,6,7]. Moreover, HA-based hydrogels can be considered a potential implantable biomaterial for soft tissue augmentation or replacement [8]. Concentrated HA aqueous solutions are characterized by the presence of a self-aggregate polymer network in which intramolecular and intermolecular interactions are present due to the establishment of hydrogen bonds and hydrophobic interactions. [9]. When the material undergoes a shear stress, the so-formed physical network confers to these solutions the properties of a viscoelastic solid, if the strain or the time of application are small enough. Nevertheless, the weak intra- and interchain interactions are not able to maintain the structure upon prolonged stress, which causes the disentanglement of the network and the flow of the solution, which behaves as a viscoelastic fluid. In this frame, to improve the performance of the material as a filler, the chemical crosslinking of HA is necessary in order to increase the rigidity of the polymer network, extend its permanence in the site of application, and reduce its susceptibility to enzymatic degradation [10]. Several crosslinkers have been used to reticulate HA, such as 1,4-Butanediol diglycidyl ether (BDDE), 1,2,7,8-diepoxyoctane (DEO), divinyl sulfone (DVS), hexamethylenediamine (HMDA), and polyethylene glycol diglycidyl ether (PEGDE) [11]. A frequently used method today for crosslinking HA is the reaction with BDDE under alkaline conditions to yield a stable covalent ether linkage between HA and the cross-linker [12,13]. Further studies have evaluated the opportunity to prepare composite hydrogels by crosslinking HA with other polymers in order to obtain hydrogels with enhanced performances. For example, HA crosslinked with lactate-modified chitosan was observed to increase the elasticity of the material, because of the electrostatic interaction between the two polymers [14]. In another work, BDDE was used to crosslink HA and bacterial cellulose to obtain a wound dress with improved surface properties and mechanical and thermal resistance [15]. Indeed, the use of cellulose-based materials is also advantageous in the preparation of biomedical devices with a structural function, such as scaffolds and fillers. Cellulose is a linear polysaccharide of glucose, linked by β-(1,4)-glycosidic bonds, that has a structural task in plants. Cellulose is biocompatible and widely abundant in nature, and is stable in a physiological environment because amylases present in animals are not able to hydrolyze β-glycosidic bonds. At the same time, despite cellulose not being intrinsically hydrophobic, it is almost insoluble in water owing to the high degree of crystallinity [16]. This hindrance can be overcome by means of the chemical modification of the hydroxyl groups of cellulose, such as etherification or esterification, in order to make the polymer soluble in water and enlarge its field of application. Many water-soluble derivatives of cellulose are currently on the market, among which the most widespread is carboxymethyl cellulose (CMC). CMC’s cellulose-derived polysaccharide is available in high-purity forms and has found, since it is FDA-approved, several biomedical applications due to its biocompatibility and low cost. CMC has a plant origin, which represents a key advantage over other natural fillers of animal origin, such as collagen, since it is less likely to elicit an immune response. Furthermore, the absence of the cellulase enzyme in humans, which digests cellulose, allows for adequate mechanical stability of CMC in vivo, compared to other natural biomaterial fillers that are susceptible to enzymatic activity. [17]. Several commercially available fillers such as Laresse, Radiesse, and Sculptra incorporate CMC with other materials, such as poly(ethylene oxide), hydroxyapatite, and poly(lactic acid). Most of these fillers use non-cross-linked formulations of CMC, which can potentially reduce their mechanical stability and in vivo retention time. Furthermore, the presence of synthetic components in these fillers can make selective removal following adverse reactions, migration, or placement very challenging, requiring invasive surgical procedures [18]. However, the right compromise between viscoelastic properties, the persistence of fillers, and biocompatibility must be achieved. The combination of HA and CMC is expected to improve CMC as a biomaterial while increasing HA’s mechanical properties and their thermal stability. In this frame, a composite hydrogel based of HA/CMC crosslinked with BDDE has been developed for soft tissue augmentation for the first time with the main aim to obtain a better-performing HA-based hydrogel filler. HA/CMC composite hydrogels were developed at different ratios and their viscoelastic properties, thermal stability, and swelling ratio were investigated in order to optimize composition and reaction conditions. The morphology and the biocompatibility response of the hydrogel that possessed the best performance were then investigated.

## 2. Results and Discussion

### 2.1. The Design of the Protocol

**Figure 1:**
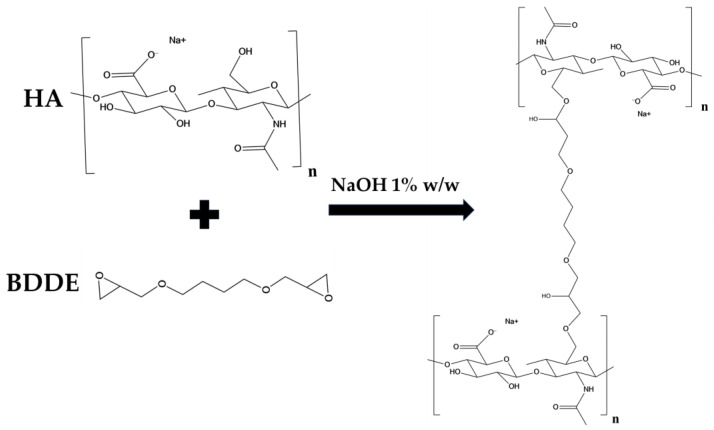
Scheme of the crosslinking reaction of HA by means of BDDE.

In this work a protocol was developed for the preparation of a HA/CMC composite hydrogel, crosslinked with BDDE, for application in soft tissue augmentation. To achieve this goal, two protocols for crosslinking HA with the same concentration of HA and BDDE were compared, investigating the effect of time and temperature (Table 1) [19]. Briefly, HA was dissolved in a NaOH 1% *w*/*w* solution, then BDDE was added and the mixture was left to react at 25 °C for 24 h and at 50 °C for 2 h, respectively. The materials prepared with the two protocols were labeled HA1 and HA2. In alkaline solution, the crosslinking of HA occurs by means of the nucleophilic addition of the primary hydroxyl groups present at the C-6 position on the N-acetyl-D-glucosamine to the epoxydic groups of BDDE, forming an ether bond (Figure 1) [20].

**Figure 2:**
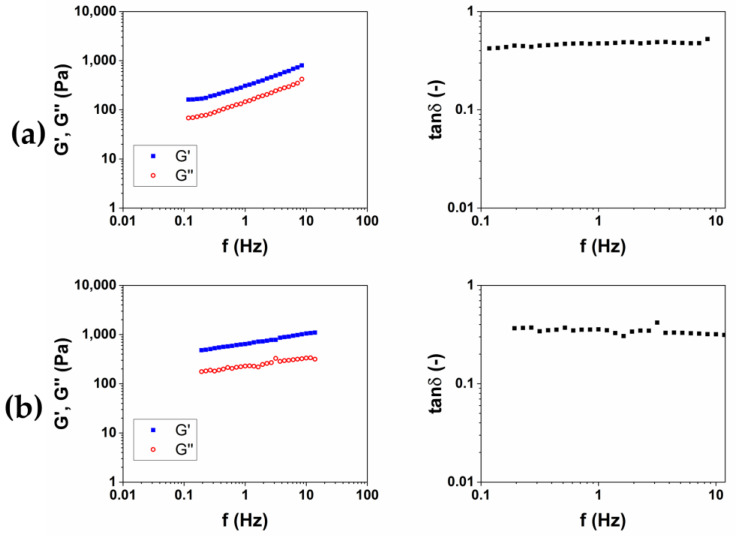
Representative images of mechanical spectra and tanδ of HA-based hydrogels crosslinked with BDDE (HA1) at 25 °C for 24 h (**a**) and (HA2) at 50 °C for 2 h (**b**).

After, for each protocol, the material obtained was put in 200 mL of bi-distilled water for three days, in order to remove the unreacted BDDE, which is known for being toxic [21]. During this purification process, each hydrogel was periodically removed, dried from excess water and weighed, in order to calculate the swelling ratio (SR) (Table 1). For all of the hydrogels examined in this work, the equilibrium was reached after 24 h. After the purification was completed, the viscoelastic moduli of the two materials were measured. The storage modulus G′, the loss modulus G″ and the loss factor tanδ as a function of the frequency were reported in Figure 2. G′ and G″ represent the elastic and viscous response of the material, respectively, and their ratio tanδ expresses the behavior of the viscoelastic materials.

**Figure 3:**
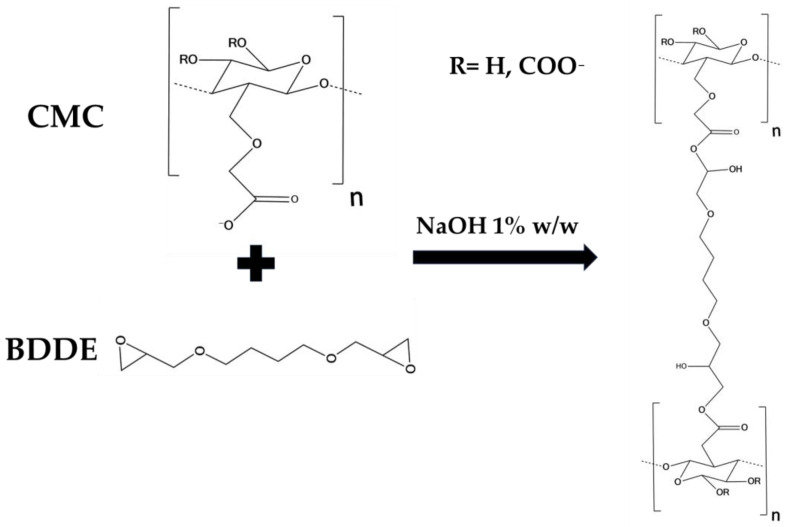
Scheme of the crosslinking reaction of CMC by means of BDDE.

**Figure 4:**
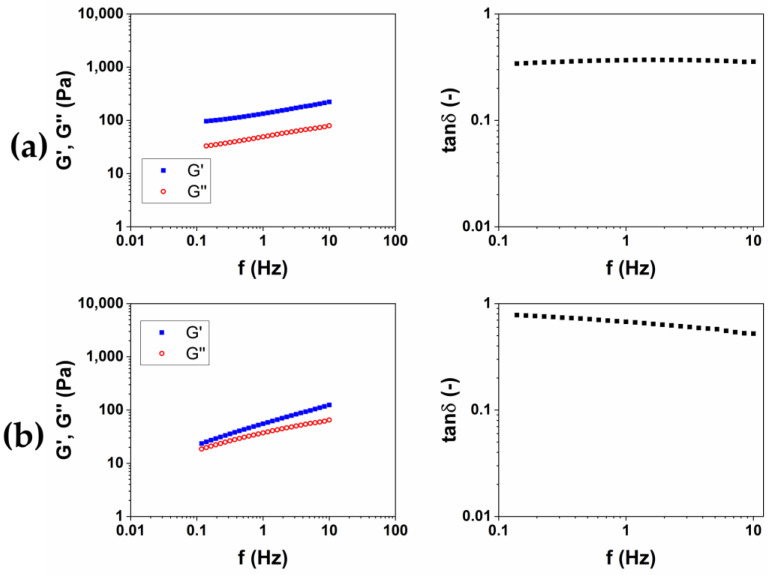
Representative images of mechanical spectra and tanδ of CMC-based hydrogels crosslinked with BDDE (CMC1) at 25 °C for 24 h (**a**) and (CMC2) at 50 °C for 2 h (**b**).

The results of rheological analysis are expressed in terms of the value of the storage modulus G′ and tanδ at 1 and 10 Hz as reported in Table 2 Both the materials exhibit a gel-like behavior, for which G′ > G″ and tanδ < 1 in all of the frequency ranges investigated. The quantitative analysis shows how the HA2 hydrogel, prepared at 50 °C for 2 h, exhibits higher values of the viscoelastic modulus G′ (640 ± 40 at 1 Hz and 1050 ± 40 at 10 Hz), lower values of tanδ (0.4 at 1 Hz and 0.32 at 10 Hz) and lower value of SR (54 *w*/*w*), compared to HA1 hydrogels. In order to evaluate the best protocol to prepare HA/CMC composite hydrogels, CMC-based hydrogels, crosslinked with BDDE, were prepared with the two methods reported in Table 3. CMC is able to form hydrogels with BDDE (Figure 3) by reacting with its carboxylate groups, which act as nucleophiles [22]. Representative mechanical spectra of the two hydrogels, labeled CMC1 and CMC2, respectively, are reported in Figure 4.

Differently from HA-based hydrogels, a higher stiffness is observed for the hydrogel CMC1, prepared at 25 °C for 24 h. Furthermore, the mechanical spectrum of the hydrogel CMC2, obtained at 50 °C for 2 h, almost does not present a trend of the moduli typical of a hydrogel (Table 2). Contrariwise, a rising trend of the viscoelastic moduli is observed (Figure 4b), and it appears that an intersection of the curves could be present for lower values of frequency. This mechanical behavior is more assimilable to entanglement polymer solutions than a hydrogel [23]. Also, the SR calculated for CMC2 was lower (18 *w*/*w*) compared to CMC1 (90 *w*/*w*), despite it being a stiffer hydrogel. It follows that, for CMC, reacting for short times at a higher temperature could not lead to a crosslinking of the material to form a hydrogel. For this reason, the protocol at 25 °C for 24 h was chosen for the preparation of the HA/CMC composite hydrogels.

### 2.2. The Optimization of the Composition

The HA/CMC composite hydrogels were prepared following the same procedure used for HA-based and CMC-based hydrogels. As reported in Table 4, hydrogels prepared with the HA/CMC weight ratios of 1:3, 1:1, and 3:1, named HCM1, HCM2, and HCM3, respectively, were examined.

**Figure 5:**
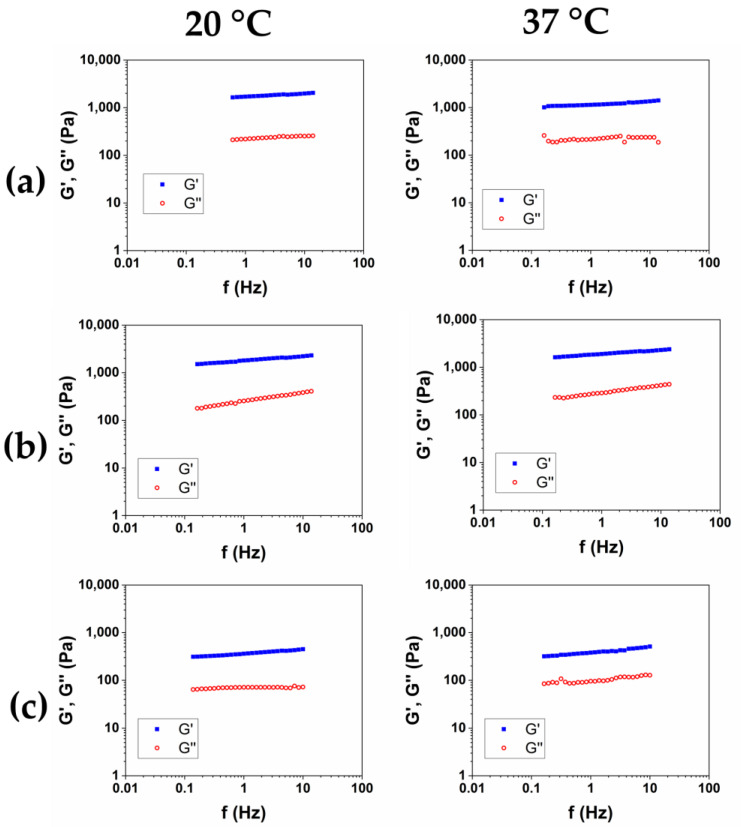
Representative mechanical spectra of HCM1 (**a**), HCM2 (**b**), and HCM3 (**c**) at 20 °C and 37 °C.

After the purification was completed, and SR was assessed, in order to investigate the viscoelastic properties of the hydrogels, the Frequency Sweep (FS) tests were performed at 20 °C and 37 °C on the three samples. The results of the frequency sweep tests of the HCM1, HCM2, and HCM3 were reported in Figure 5a, b, and c, respectively. The mechanical spectra were analyzed in terms of the dependence of the storage modulus G′ and of the loss factor tanδ as a function of the frequency for the two temperatures (Table 5). All of the HA/CMC-based materials exhibit a trend of the viscoelastic moduli and tanδ proper of the hydrogels. The data collected at 20 °C and 37 °C was nearly overlapping, and the irrelevant effects of temperature were observed. The composite hydrogels HCM1 and HCM2 exhibited higher viscoelastic moduli and lower loss factor, compared to the HA1 hydrogel, at the same overall polymer concentration. HA and CMC appear, therefore, to form a synergical network with an increase of rheological properties after the crosslinking. In particular, the hydrogel HCM2, prepared with an HA/CMC weight ratio of 1/1, is the hydrogel with the higher values of the moduli and simultaneously higher SR. Finally, according to the data collected for the CMC-based hydrogels, the sample HCM3, with the highest content of CMC, was the hydrogel with the lowest mechanical properties among the three prepared.

**Figure 6:**
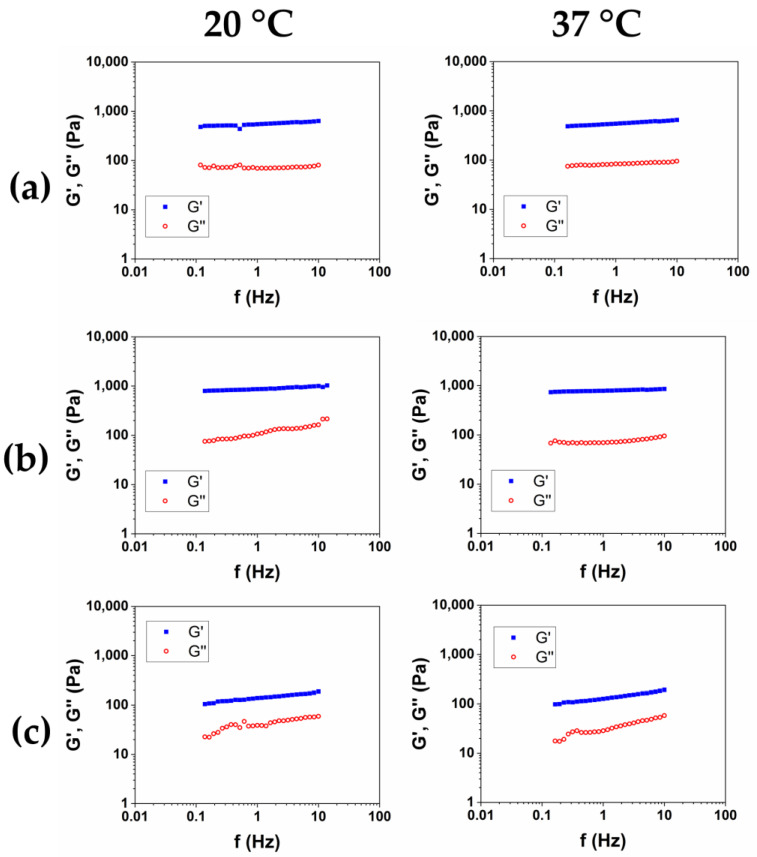
Mechanical spectra of HCM1 (**a**), HCM2 (**b**), and HCM3 (**c**) after sterilization in an autoclave at 20 °C and 37 °C.

In order to obtain a product suitable for withstanding high-impact treatments like, for example, thermal sterilization, the rheological characterization of the hydrogels has been completed by measuring the viscoelastic moduli as a function of the frequency, at 20 °C and 37 °C, after sterilization in an autoclave (AC) at 121 °C for 20 min. The representative mechanical spectra of the three autoclaved hydrogels were reported in Figure 6a–c, and the rheological properties were reported in Table 6.

Among the three hydrogels, after the autoclave sterilization, HCM2 has the highest storage modulus, and the lower value of tanδ. Furthermore, compared to the material that was not autoclaved, expressed as G′/G′_AC_, HCM2 has the lower loss of mechanical properties at 20 °C, while at 37 °C the loss of mechanical properties was comparable, probably because of the thermal degradation, which caused a reduction in the structuration of the materials.

### 2.3. Fourier-Transformed Infrared (FT-IR) Analysis

**Figure 7:**
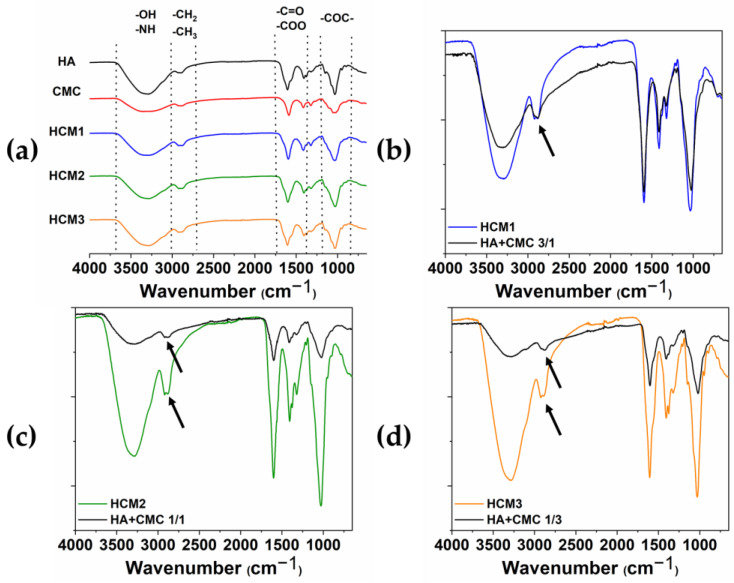
ATR-FTIR spectra of HA, CMC, HMC1, HMC2 and HMC3 (**a**); HCM1 and HA/CMC 3/1 before adding BDDE (**b**); HCM2 and HA/CMC 1/1 before adding BDDE (**c**); and HCM3 and HA/CMC 1/3 before adding BDDE (**d**). The arrows highlighted the changes in shape and intensity of the peak associated with the stretching of –CH_2_ and –CH_3_ after the crosslinking with BDDE.

The chemical modification of HA/CMC composite hydrogels has been confirmed by FTIR. The ATR-FTIR spectra acquired from the various samples HCM1, HCM2, and HCM3 and the single components of the native HA and CMC are shown in Figure 7a, along with the comparison between HCM1 and HA/CMC 3/1 (b), HCM2 and HA/CMC 1/1 (c), and HCM3 and HA/CMC 1/3 (d) before and after the addition of BDDE. It can be possible to identify the typical polysaccharide –OH signals at the region between the 3000 cm^−1^–3700 cm^−1^ [24,25]. The peaks in the region between the 3000 cm^−1^ and 2700 cm^−1^ are associated with the stretching of –CH_2_ and –CH_3_, while the peaks at 1600 cm^−1^ and 1412 cm^−1^ are associated with the symmetric and asymmetric stretching of –COO groups. Finally, at 1030 cm^−1^ the C-O-C symmetric stretching ether bands are observed. The comparison between the spectra of the polymers mixtures after and before the addition of the crosslinking agent BDDE provided information about the chemical modification occurring in the composite hydrogels. Indeed, the peak associated with the stretching of –CH_2_ and –CH_3_ changes in shape and intensity after the crosslinking with BDDE occurs. Moreover, the presence of the BDDE covalently cross-linked to polysaccharides is suggested by the absence of the peaks 1256 cm^−1^ and 908 cm^−1^ that, according to the literature, belong to the asymmetric and symmetric stretching vibrations of the epoxy groups of BDDE [26].

### 2.4. Morphological SEM Evaluation

**Figure 8:**
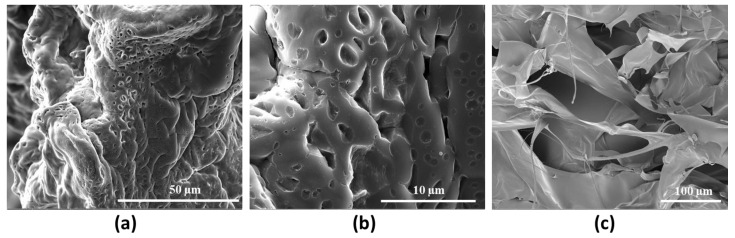
Representative SEM images of HCM2 at (**a**) 3000× and (**b**) 12,000× magnification and its cross section 800× (**c**) magnification.

The morphology of the selected hydrogel HCM2, with higher mechanical performance, was qualitatively investigated by means of Scanning Electron Microscopy analysis (Figure 8). The images acquired show the dense crosslinking networks responsible for the rheological properties and for the thermal stability of the hydrogel. In particular, Figure 8c shows information about the cross section of the HCM2 gels, in which it is possible to observe the porosity and scaffold interconnection [27].

### 2.5. Biological Response

**Figure 9:**
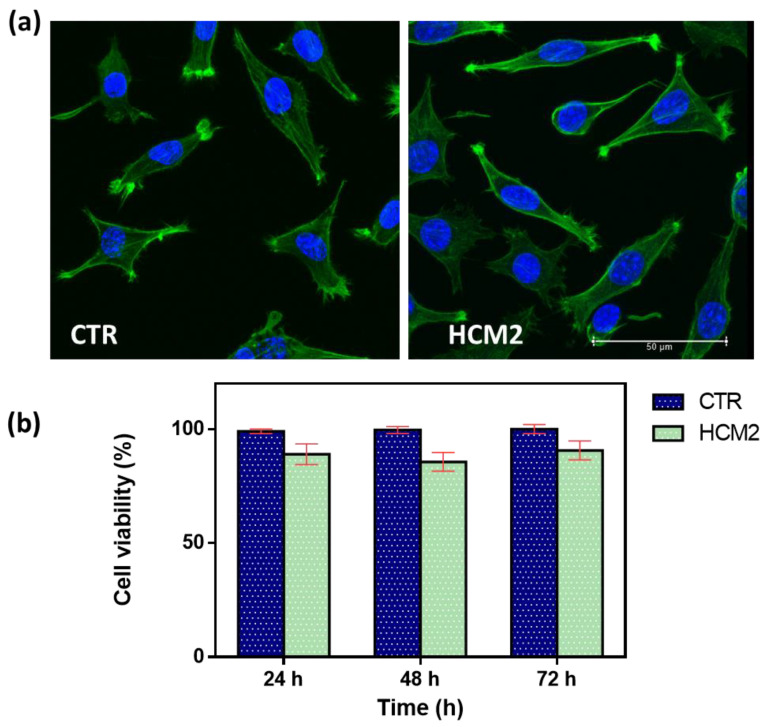
(**a**) Representative cell morphology of control L929 fibroblast cells and L929 after 24 h of incubation with the selected HCM2 hydrogels. Actin filaments, stained with phalloidin-FITC (green), and DAPI (blue)-stained nuclei cells. Images were acquired with a resolution of 1024 × 1024 pixels with a 63× oil immersion objective. (**b**) Cell viability percentage (%) of the L929 cells, after incubation at 24, 48, and 72 h compared to the control untreated cells. The data are representative of three repeated experiments in triplicate.

In vitro biological response in terms of the cell viability percentage and morphological analysis of cells represents a key feature in evaluating the design of hydrogels useful as fillers in soft tissue augmentation applications. The first safety assessment has been investigated for the selected hydrogel HCM2, which possessed the best performance properties in terms of rheological properties and swelling. It has been widely established that the viscoelastic characteristics of the materials influence cell behavior, affecting the biocompatibility of the cells [28,29]. Biocompatibility results were first assessed via cell morphology (Figure 9a). Actin filaments, a constituent of the cytoskeleton of the cells, were stained with FITC phalloidin after 24 h of incubation with HCM2 hydrogel and in untreated control. Both treated and untreated L929 cells samples exhibited a typical non-cytotoxic fibroblast-like morphology [30]. The quantitative analysis of L929 cell viability percentage has been evaluated using an Alamar blue (AB) assay as reported in Figure 9b. The HCM2 hydrogels showed good safety after 24, 48, and 72 h of incubation with L929 cells, compared to the untreated cells control. In particular, after 24, 48, and 72 h of the incubation of HCM2, L929 cell viability percentage is around 90%, indicating, in accordance with the ISO 10993–5: 2009 standards [31], the good biocompatibility of the hydrogels. Indeed, these standards specify that cell viabilities greater than 70% indicate the non-cytotoxic behavior of tested biomaterials, thus, suggesting the absence of the toxic BDDE residues in the hydrogels [32]. Overall, these results indicated that the HCM2 hydrogels have good biocompatibility properties, indicating that the purification process of the HCM2 gel following crosslinking by means of BDDE was successfully achieved. In fact, all of the unreacted crosslinker, which is notoriously cytotoxic, appears to have been removed during the swelling process.

## 3. Conclusions

HA-based fillers are widely employed in soft tissue augmentation, both in cosmetic and in chirurgical practice, but the performance of HA-based devices is often affected by low mechanical properties and poor resistance to sterilization processes. In this work, for the first time, HA/CMC composite hydrogels crosslinked with BDDE were prepared. The optimal conditions in terms of operative temperature and reaction time to crosslink HA and CMC separately were evaluated with a rheological analysis of the hydrogels. The results suggested that the better conditions for reticulating HA and CMC were 25 °C for 24 h. Then, hydrogels with a different HA/CMC ratio were prepared, and their rheological properties were investigated before and after sterilization in an autoclave at 121 °C for 20 min. The hydrogel with an HA/CMC ratio of 1/1, labeled HCM2, exhibited the highest values of the viscoelastic moduli before and after the thermal treatment. The FTIR has given us information about the occurrence of chemical modification by means of BDDE in the composite hydrogels. The morphological analysis of HCM2 via SEM highlighted the densely reticulate structure of the hydrogel. Finally, the biocompatibility response, as shown by incubating HCM2 on L929 cell fibroblast, indicated a good cell viability percentage of around 90% at 24, 48, and 72 h and along with the morphological analysis, showed the overall success of the purification process by washing. However, significant research gaps remain, including a lack of long-term in vivo investigations and immune- toxicity evaluations. Further work is needed to understand the practical applications of these HA hydrogel composites. Nevertheless, the use of CMC resulted in improvements in the HA-based hydrogel properties, indicating that HA/CMC composite hydrogels cross-linked with BDDE represent a promising platform for the design of filler implants in soft tissue augmentation.

## 4. Materials and Methods

### 4.1. Hydrogel Preparation

Hyaluronic acid (803 KDa) and CMC (750 KDa) were kindly provided by Altergon s.r.l (Morra De Sanctis, Italy), BDDE was purchased from Sigma Aldrich. Two protocols for the crosslinking of HA with BDDE were evaluated as a starting point for the preparation of the hydrogels [19]. The conditions employed were reported in Table 1 and Table 2, and were used to prepare HA/CMC-based hydrogels at different polymer ratios. According to the literature, the use of a high polymer concentration is necessary to obtain HA-based covalently crosslinked hydrogels [33,34]. HA and CMC hydrogels were also prepared as a reference. Briefly, for each protocol NaOH 1% *w*/*w* solution was gradually added to dry polymer powder, and the mixture was gently stirred with a spatula, in order to promote polymer hydration and avoid the formation of bubbles. When the addition of NaOH solution was complete, the mixture was left for 24 h at room temperature, after that it appeared as homogeneous systems. Then, 25 µL of BDDE were added to the mixture, which was left reacting for the time required for each protocol (2 h at 50 °C or 24 h at RT). Once the time had passed, the mixture was neutralized by adding HCl, and the hydrogels were put in 200 mL of water for 3 days to remove unreacted BDDE.

### 4.2. Swelling Ratio

In order to evaluate the swelling ratio (SR) of each hydrogel prepared, during the purification the hydrogels were withdrawn periodically, dried of excess water, and weighed until the mass was stable over time. For all of the hydrogels the equilibrium was reached after 24 h. After purification was completed, the hydrogels were frozen at T = −80 °C and freeze dried. The dry mass was then weighed, and the swelling ratio was calculated according to Equation (1):

(1) $$SR=\frac{W_{h}-W_{d}}{W_{d}}$$

where *W_h_* is the mass of the hydrogel at the equilibrium and *W_d_* is the mass of the dry hydrogel.

### 4.3. Rheological Analysis

The viscoelastic properties of the hydrogels were evaluated by means of oscillatory regime tests using a rotational rheometer Haake Mars III (Thermo Fisher Scientific, Waltham, MA, USA) equipped with a parallel plate geometry, 35 mm plate diameter, 0.5 mm gap, and a thermostatic bath. Hydrogels are viscoelastic materials, and their mechanical response to a shear stress τ presents elements of an elastic solid (Hooke’s law, Equation (2)) and of a Newtonian liquid (Newton’s law, Equation (3)):

(2) τ = Gγ

(3) τ = η dγ/dt

where G is the elastic modulus of the solid, η is the dynamic viscosity of the liquid and γ is the imposed deformation. When a sinusoidal strain with oscillatory frequency ω is exerted over time, the response of the elastic solid (Equation (3)) results shifted by 90° compared to that of the ideal fluid Equation (4). The frequency ω is the oscillation frequency in s^−1^, which can also be reported as 2πf, where f is in Hz.

(4) $$\tau =G\gamma_{0}\sin\left(\omega t\right)$$

The mechanical response of a viscoelastic material under a sinusoidal stress can be therefore written as in Equation (5):

(5) $$\tau ={G^{*}\gamma }_{0}\sin\left(\omega t+\delta \right)$$

where G* is the complex modulus of the material, γ_0_ is the amplitude of the strain and δ is the shift with respect to the behavior of the ideal solid, and is comprised between 0° and 90°. Applying the sum sin identity, Equation (6) can be written as:

(6) $$\tau ={G^{*}\gamma }_{0}\sin\left(\omega t\right)\cos\left(\delta \right)+{G^{*}\gamma }_{0}\cos\left(\omega t\right)\sin(\delta )$$

Equation (6) expresses the response of a viscoelastic material under sinusoidal strain as the sum of two contributions, one in phase with the strain, and one shifted by 90. Inside the equation it is possible to define two viscoelastic moduli (Equations (7) and (8)):

(7) $$G^{\prime }=G^{*}\cos\left(\delta \right)$$

(8) $$G^{\prime \prime }=G^{*}\sin(\delta )$$

where G′ is the storage modulus, and expresses the elastic response of the material, and G″ is the loss modulus, and expresses the viscous response of the material. Oscillatory tests allow, therefore, to break down into two contributions the mechanical response of a viscoelastic material.

In this work frequency sweep (FS) tests were performed to measure the viscoelastic moduli G′ and G″ as a function of frequency in the range 0.1–13 Hz at a fixed strain of 0.5%. The value of the strain was chosen to have a linear viscoelastic response, independent on the strain itself, and was determined by means of strain sweep tests. Form FS tests the ratio between G″ and G′, the loss factor tan δ (Equation (9)) was calculated as follows:

(9) $$tan\delta =\frac{G^{\prime \prime }}{G^{\prime }}$$

tan δ expresses the ratio between the viscous and the solid ratio of the material [35].

Rheological analysis was also performed in order to evaluate the thermal stability of the hydrogels. The hydrogels were treated in an autoclave for 20 min at 121 °C, and FS tests were carried out on the heat-treated hydrogels. The reduction in mechanical properties was expressed for each material by dividing G′ at 1 Hz before the autoclave for G′ at 1 Hz after the autoclave.

### 4.4. Fourier-Transformed Infrared (FT-IR) Analysis

Portions from HA and CMC hydrogel and cross-linked composite hydrogels were obtained and characterized using Perkin Elmer Frontier Fourier Transform Infrared Spectroscopy FT-IR (Waltham, MA, USA), with a single-reflection, universal ATR-IR accessory. All spectra were recorded between 4000 and 650 cm^−1^ with a resolution of 4 cm^−1^ and the data were manipulated using OriginPro 2018 software.

### 4.5. Morphological Analysis

In order to obtain qualitative morphological information, scanning electron microscopy (Quanta 200 FEG, FEI Company, Hillsboro, OR, USA) was employed. The samples were lyophilized and platinum/palladium–sputtered to perform the analysis.

### 4.6. Biological Resposnse

#### 4.6.1. Cell Culture

In order to evaluate the biological response of the HA/CMC composite hydrogels, mouse fibroblast L929 cells derived from mouse C34/An connective tissues (Sigma-Aldrich, Burlington, MA, USA) were grown in a T-75 cell culture flask (VWR, Radnor, PA, USA) at 37 °C and 5% CO_2_. Cell culture medium Dulbecco’s Modified Eagle’s medium (DMEM) (Microgem, Naples, Italy) supplemented with 10% of fetal bovine serum and antibiotics (penicillin G sodium 100 U/mL, streptomycin 100 μg/mL) were used and changed every 3–4 days.

#### 4.6.2. Cell Viability and Morphological Assay

To assess the cell morphology, L929 cells were seeded at a density of 1 × 10^4^ cells/mL on fluorodish-35 mm (VWR, Radnor, PA, USA). The selected hydrogel HCM2 was sectioned and deposited in 3 wells of a 24 well plate and UV sterilization was carried out at 284 nm for 30 min. Subsequently, the DMEM was added until the samples were covered: these were left in an incubator at 37 °C for 24 h. The cells were incubated with the hydrogel eluate for 24 h. Then, the samples were washed three times with PBS and fixed with 10% formaldehyde for 1 h at 4 °C. Cells were permeabilized with Triton X-100 0.1% in PBS for 3–5 min. The actin filaments were stained with FITC phalloidin/PBS for 30 min at RT. Finally, after two washes with PBS to remove the unbound phalloidin, cell nuclei were stained with 4′,6-diamidino-2-phenylindole DAPI, (Sigma-Aldrich). The samples were observed using a confocal microscope system (Leica TCS SP8) with a 63× oil immersion objective. Images were acquired with a resolution of 1024 × 1024 pixels.

In order to study the cell viability, a density of 5 × 10^3^ cells/mL of L929 cells were seeded on a 96-well plate (World Precision Instruments, Inc., Sarasota, FL, USA). The cells were then incubated for 24, 48, and 72 h with 200 µL of the HCM2 eluate. Then, the Alamar blue assay (AB) was performed by adding AB reagent, at 10% *v*/*v* with respect to the medium to the samples and incubated at 37 °C for 4 h. The absorbance of the samples was measured using a spectrophotometer plate reader (Multilabel Counter, 1420 Victor, Perkin Elmer, Waltham, MA, USA) at 570 nm and 600 nm. The AB reagent dye indicates an oxidation-reduction by changing color in response to the chemical reduction in the growth medium, resulting from cell viability. Data are expressed as the percentage difference between treated and control to evaluate the percentage of reduction (Reduction %), which is calculated with the following formula (Equation (10)):

(10) $$Reduction\;\left(\%\right)=\frac{\left(O_{2}\times A_{1}\right)-(O_{1}\times A_{2})}{\left(O_{2}\times P_{1}\right)-(O_{1}\times P_{2})}\times 100$$

where *O*_1_ is the molar extinction coefficient (*E*) of oxidized AB at 570 nm; *O*_2_ is the *E* of oxidized AB at 600 nm; *A*_1_ is the absorbance of test wells at 570 nm; *A*_2_ is the absorbance of test wells at 600 nm; *P*_1_ is the absorbance of the control well at 570 nm; and *P*_2_ is the absorbance of the control well at 600 nm. The percentage of reduction for each sample was normalized to the percentage of reduction for the control to obtain the cell viability percentage.

## Figures and Tables

**Table 1:** Experimental conditions for the preparation of HA hydrogels.

Sample	C HA (mg/mL)	C BDDE (µL/mL)	NaOH%	T, °C	Time, h	SR (*w*/*w*)
HA1	133.3	8.33	1	25	24	80
HA2	133.3	8.33	1	50	2	54

**Table 2:** Storage modulus G′ and loss factor of the HA-based and CMC-based hydrogels.

Sample	1 Hz		10 Hz	
	G′ (Pa)	tanδ (-)	G′ (Pa)	tanδ (-)
HA1	310 ± 30	0.5 ± 0.02	420 ± 30	0.52 ± 0.02
HA2	640 ± 40	0.4 ± 0.02	1050 ± 40	0.32 ± 0.02
CMC1	130 ± 30	0.4 ± 0.02	220 ± 30	0.35 ± 0.02
CMC2	55 ± 30	0.7 ± 0.02	120 ± 30	0.52 ± 0.02

**Table 3:** Experimental conditions for the preparation of CMC hydrogels.

Sample	C CMC (mg/mL)	C BDDE (µL/mL)	NaOH%	T, °C	Time, h	SR (*w*/*w*)
CMC1	133.3	8.33	1	25	24	90
CMC2	133.3	8.33	1	50	2	18

**Table 4:** Composition, protocol and swelling ratio (SR) of the HA/CMC composite hydrogels prepared.

Sample	HA/CMC Weight Ratio	C HA (mg/mL)	C CMC (mg/mL)	C BDDE (µL/mL)	T (°C),	Time (h)	SR (*w*/*w*)
HCM1	3/1	100	33.3	8.33	25	24	58
HCM2	1/1	66.6	66.6	8.33	25	24	76
HCM3	1/3	33.3	100	8.33	25	24	50

**Table 5:** Viscoelastic properties of the HA/CMC composite hydrogels measured at 20 °C and 37 °C.

Sample	20 °C, 1 Hz		20 °C, 10 Hz		37 °C, 1 Hz		37 °C, 10 Hz
	G′ (Pa)	tanδ (-)	G′ (Pa)	tanδ (-)	G′ (Pa)	tanδ (-)	G′ (Pa)
HCM1	1700 ± 130	0.13 ± 0.03	2000 ± 120	0.13 ± 0.02	1100 ± 200	0.19	1400 ± 200
HCM2	1900 ± 100	0.15 ± 0.05	2300 ± 100	0.2 ± 0.04	1800.0 ± 200	0.14	2200 ± 100
HCM3	380 ± 90	0.25 ± 0.05	510 ± 150	0.25 ± 0.03	360 ± 120	0.2	500 ± 100

**Table 6:** Viscoelastic properties of the HA/CMC hydrogels after sterilization in an autoclave, measured at 20 °C and 37 °C.

Sample	20 °C, 1 Hz			37 °C, 1 Hz		
	G′ (Pa)	tanδ (-)	G′/G′_AC_ (-)	G′ (Pa)	tanδ (-)	G′/G′_AC_ (-)
HCM1	550 ± 90	0.15 ± 0.03	3.1	550 ± 110	0.13 ± 0.04	2.1
HCM2	870 ± 120	0.13 ± 0.05	2.2	800 ± 150	0.09 ± 0.02	2.3
HCM3	140 ± 90	0.30 ± 0.02	2.7	190 ± 90	0.30 ± 0.03	1.9

## Data Availability

The data presented in this study are openly available in article.
